# Platelet SR-PSOX/CXCL16–CXCR6 Axis Influences Thrombotic Propensity and Prognosis in Coronary Artery Disease

**DOI:** 10.3390/ijms231911066

**Published:** 2022-09-21

**Authors:** Tianyun Guan, Frederic Emschermann, Christoph Schories, Patrick Groga-Bada, Peter Martus, Oliver Borst, Meinrad Gawaz, Tobias Geisler, Dominik Rath, Madhumita Chatterjee

**Affiliations:** 1 Department of Cardiology and Angiology, University Hospital Tübingen, Otfried Müller Straße 10, 72076 Tübingen, Germany; 2 Institute for Clinical Epidemiology and Applied Biostatistics, University Hospital Tübingen, 72076 Tübingen, Germany; 3 Department of Pharmacology, Experimental Therapy and Toxicology, University Hospital Tübingen, Wilhelmstrasse 56, 72074 Tübingen, Germany

**Keywords:** platelet, CXCL16, CXCR6, coronary artery disease, prognosis

## Abstract

Platelets express the transmembrane chemokine SR-PSOX/CXCL16, proteolytic cleavage of which generates the sCXCL16 soluble-(s) chemokine. The sCXCL16 engages CXCR6 on platelets to synergistically propagate degranulation, aggregation and thrombotic response. Currently, we have investigated the pro-thrombotic and prognostic association of platelet CXCL16–CXCR6 axis in CAD-(*n* = 240; CCS *n* = 62; ACS *n* = 178) patients. Platelet surface-associated-CXCL16 and CXCR6 surface expression ascertained by flow cytometry correlated significantly with platelet activation markers (CD62P denoting degranulation and PAC-1 binding denoting α_2b_β_3_-integrin activation). Higher platelet CXCL16 surface association (1st quartile vs. 2nd–4th quartiles) corresponded to significantly elevated collagen-induced platelet aggregation assessed by whole blood impedance aggregometry. Platelet-CXCL16 and CXCR6 expression did not alter with dyslipidemia, triglyceride, total cholesterol, or LDL levels, but higher (>median) plasma HDL levels corresponded with decreased platelet-CXCL16 and CXCR6. Although platelet-CXCL16 and CXCR6 expression did not change significantly with or correlate with troponin I levels, they corresponded with higher Creatine Kinase-(CK) activity and progressively deteriorating left ventricular ejection fraction (LVEF) at admission. Elevated-(4th quartile) platelet-CXCL16 (*p* = 0.023) and CXCR6 (*p* = 0.030) measured at admission were significantly associated with a worse prognosis. However, after Cox-PH regression analysis, only platelet-CXCL16 was ascertained as an independent predictor for all-cause of mortality. Therefore, the platelet CXCL16–CXCR6 axis may influence thrombotic propensity and prognosis in CAD patients.

## 1. Introduction

Inflammatory chemokines in the immediate vascular microenvironment or circulation engage cognate receptors on platelets. They influence thrombotic functions, and the thrombo-inflammatory potential of platelets, in interacting with the inflamed endothelium and inflammatory leukocytes [1,2]. Both cytokines and chemokines are widely investigated therapeutic targets in atherothrombotic cardio-/cerebrovascular diseases [3,4]. The IFN-γ regulated chemokine CXCL16 is a unique transmembrane scavenger receptor (SR-PSOX/CXCL16) [5] for the atherogenic mediator oxidized low-density lipoprotein (oxLDL), and the ‘eat-me’ signal phosphatidylserine (PS) exposed on the surface of apoptotic cells [6]; also procoagulant platelets [7]. A polymorphism within exon four of *CXCL16* is associated with increased coronary stenosis severity and reduced minimum luminal diameter [5]. Platelets express SR-PSOX/CXCL16 [8,9] like macrophages [10], T cells [11,12], inflamed endothelial [13], and smooth muscle cells [14,15]. Proteolytic cleavage of the extracellular domain of transmembrane-SR-PSOX/CXCL16 by metalloproteinase ADAM10 [13] generates the soluble-(s) chemokine sCXCL16, which engages its receptor CXCR6 on a variety of target cells. The sCXCL16 is richly deposited at atherosclerotic lesions [6,16,17]. Serum levels of sCXCL16 are significantly elevated in acute coronary syndrome (ACS) patients as compared to those with stable angina pectoris (SAP), although not associated with either the severity of coronary stenosis or TIMI risk score [18]. However, levels of sCXCL16 in ACS patients correlate with inflammatory and metabolic risk factors [19]. Serum sCXCL16 level influenced by pro-inflammatory hs-CRP is also suggested as a peripheral diagnostic biomarker for diabetic patients with coronary artery disease (CAD) [20]. Increased serum sCXCL16 levels, as a strong independent indicator of long-term mortality [20,21], are prognostically unfavorable in ACS patients and patients with intermediate coronary lesions [21]. Recently, in a subgroup of 5142 patients randomized in the PLATO trial (Platelet Inhibition and Patient Outcome), serum sCXCL16 levels were shown to be independently associated with cardiovascular death and morbidity in ACS patients [22]. Additionally, sCXCL16 has been suggested to provide valuable information on the risk of eventually succumbing to MI, tracked over 11.3 years among 58,761 healthy men and women who were free of cardiovascular disease when enrolled in the large population-based HUNT cohort [23]. The diagnostic and prognostic significance of sCXCL16, taken together, suggests its potential to aid risk stratification in cardiovascular disease patients. Since platelets express transmembrane-SR-PSOX/CXCL16 [9] and may contribute to serum sCXCL16, we have extended these clinical findings by exploring the relevance of platelet SR-PSOX/CXCL16 and CXCR6 in influencing thrombotic potential and prognosis among CAD patients over a three-year follow-up period. Platelets express SR-PSOX/CXCL16 as transcript and protein [9]. Platelet surface expression of SR-PSOX/CXCL16 is increased upon activation with agonists-ADP, TRAP, and oxLDL and assists in oxLDL binding to TRAP-activated platelets [9]. This is of particular significance since platelets from ACS patients show increased surface-associated [24] and intraplatelet oxLDL [25] levels, acquisition of which might be assisted by SR-PSOX/CXCL16 acting as a scavenger receptor. Previously, in a small cohort of 61 patients undergoing coronary angiography, we observed significantly enhanced platelet SR-PSOX/CXCL16 surface expression among ACS patients compared to those with SAP [9], which correlated with levels of pro-inflammatory hsCRP and CK.

Subsequently, we reported the expression of CXCR6, the receptor for sCXCL16, in platelets at transcript and protein levels. Moreover, we have demonstrated that CXCR6 ligation by recombinant sCXCL16 triggers the activatory PI3k-Akt, signaling cascade to instigate degranulation, α_2b_β_3_-integrin activation, and synergistically stimulating aggregatory response to ADP. Recombinant sCXCL16 substantiates platelet adhesion to endothelial monolayer in vitro and to the injured carotid artery of mice in vivo; effects that are significantly ablated in mice deficient in CXCR6, Akt1 and Akt2. Counteraction provided by apyrase and P2Y12-antagonist clopidogrel suggests the involvement of ADP in sCXCL16–CXCR6 axis mediated synergistic effects on platelet functions [8] and plausible clinical intervention through anti-platelet therapy. In a more pathophysiological setting, immobilized sCXCL16 richly deposited at atherosclerotic lesions or on TNF-α, IFN-γ-inflamed human radial artery segments efficiently capture CXCR6 expressing platelets [26], inducing intraplatelet calcium mobilization and irreversible platelet aggregation [26]. In light of these experimental pieces of evidence and the recently reported prognostic significance of serum sCXCL16 in ACS patients, shown in the PLATO trial [22], the current investigation expands our previous observation on the platelet CXCL16–CXCR6 axis and explores its translational implication in a cohort of *n* = 240 CAD patients. Additionally, current results offer novel clinical insights on the prognostic association of platelet-CXCL16 and CXCR6.

## 2. Results

### 2.1. Platelets may Contribute to Circulatory sCXCL16 and Engage Circulatory sCXCL16

We enrolled *n* = 62 CCS, and *n* = 178 ACS patients in the current clinical cohort of CAD (*n* = 240) patients. Baseline characteristics of the enrolled patients are presented in Table 1.

**Figure 1:**
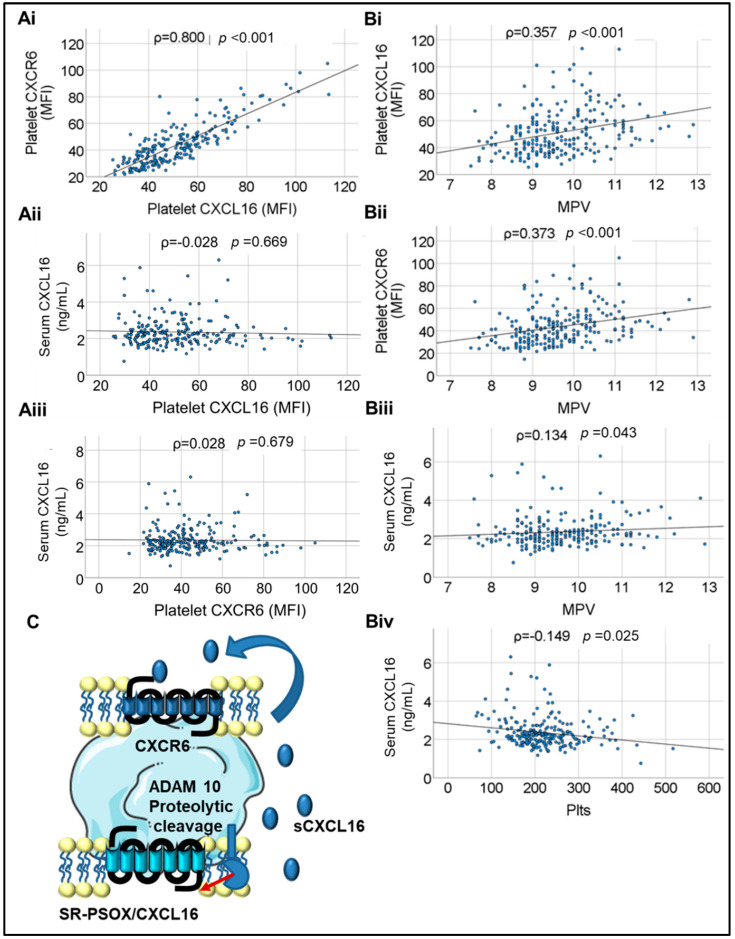
(**Ai**)-(**Aiii**)**.** Correlations between platelet surface-associated CXCL16, platelet-CXCR6, and serum levels of soluble (s)CXCL16 in CAD patients. (**Bi**)–(**Biii**). Correlations between platelet-CXCL16, platelet-CXCR6, serum levels of soluble CXCL16 in CAD patients, and Mean platelet volume (MPV). (**Biv**). Correlation between serum sCXCL16 and circulatory platelet count (Plts = Platelet count × 10^3^/µL) in CAD patients. Correlations were assessed by Spearman’s rank correlation coefficient (ρ); *p*: level of significance; MFI: mean fluorescence intensity. (**C**). Schematic diagram depicting platelets as a potential source of sCXCL16 generated by the proteolytic cleavage of the transmembrane-SR-PSOX/CXCL16 by ADAM10. The sCXCL16 may engage its cognate receptor CXCR6 on platelets to elicit pro-thrombotic effects.

Platelet surface-associated CXCL16 (platelet transmembrane-SR-PSOX/CXCL16 and platelet surface-associated circulatory sCXCL16 combined, as detected by flow cytometry and hereafter referred to as platelet-CXCL16) showed a strong and significant positive correlation with platelet surface expression of CXCR6 (ρ = 0.800, *p* < 0.001). This suggested that much of platelet surface-associated CXCL16 may be circulatory sCXCL16 sequestered by ligation to platelet CXCR6, its cognate receptor. We also evaluated serum levels of sCXCL16 (as assessed in the PLATO study [22] and HUNT cohort [23]) in CAD patients. However, neither platelet-CXCL16 nor platelet CXCR6 showed evident correlation with serum sCXCL16, denoting that serum or circulatory sCXCL16 may be derived from other cellular sources like leukocytes and the vascular endothelium, in addition to circulating platelets. Platelet-CXCL16 and CXCR6 predictably correlated significantly with mean platelet volume (MPV) (ρ = 0.357, *p* < 0.001 and ρ = 0.373, *p* < 0.001, respectively) (Figure 1A,B). Moreover, we observed a significant correlation between MPV and sCXCL16 (ρ = 0.134, *p* = 0.043), which might suggest platelets as a potential source of circulatory sCXCL16; but at the same time, a significant inverse correlation (ρ = −0.149; *p* = 0.025) between platelet count and sCXCL16 (Figure 1C) could suggest that platelets may substantially sequester sCXCL16 available in circulation.

Results from experimental in vitro studies suggest the involvement of a positive feedback loop mediated through ADP in sCXCL16-platelet CXCR6 exerted pro-thrombotic effects, which are counteracted in the presence of apyrase and P2Y_12_ antagonist [8]. Therefore, we ascertained the influence of P2Y_12_ antagonists on platelet-CXCL16, CXCR6 and serum sCXCL16 levels in the current clinical cohort. Neither ticagrelor nor clopidogrel administration showed any significant impact on platelet-CXCL16 (*p* = 0.443, *p* = 0.224, respectively) and platelet CXCR6 (*p* = 0.132, *p* = 0.070, respectively) surface expression. Among ticagrelor-administered patients, platelet-CXCL16 was median MFI (25th; 75th percentile) = 45.15 (38.00; 54.30) with ticagrelor vs. 47.65 (38.60; 58.89) *w/o* ticagrelor; CXCR6 was median MFI (25th; 75th percentile) = 35.60 (30.35; 42.65) with ticagrelor vs. 41.10 (32.20; 50.95) *w/o* ticagrelor. However, patients administered with ticagrelor showed significantly (*p* = 0.039) reduced levels of serum sCXCL16 [median (25th; 75th percentile) = 1.92 (1.66; 2.22) ng/mL with ticagrelor vs. 2.19 (1.90; 2.58) ng/mL *w/o* ticagrelor]. Similarly, among clopidogrel-administrated patients in the current cohort platelet-CXCL16 levels were median MFI (25th; 75th percentile) = 47.00 (38.30; 57.20) for *w/o* clopidogrel vs. 52.53 (39.76; 64.84) for w/clopidogrel. For platelet CXCR6, the levels were median MFI (25th; 75th percentile) = 40.00 (31.55; 48.93) for *w/o* clopidogrel vs. 46.55 (35.95; 54.50) for w/clopidogrel]. Serum levels of sCXCL16 were median (25th; 75th percentile) = 2.17 (1.85; 2.61) ng/mL for *w/o* clopidogrel vs. 2.25 (1.95; 2.50) ng/mL for w/clopidogrel and exhibited no significant differences (*p* = 0.265).

### 2.2. Platelet CXCL16–CXCR6 Axis may Influence Pro-Thrombotic Disposition in CAD Patients

**Figure 2:**
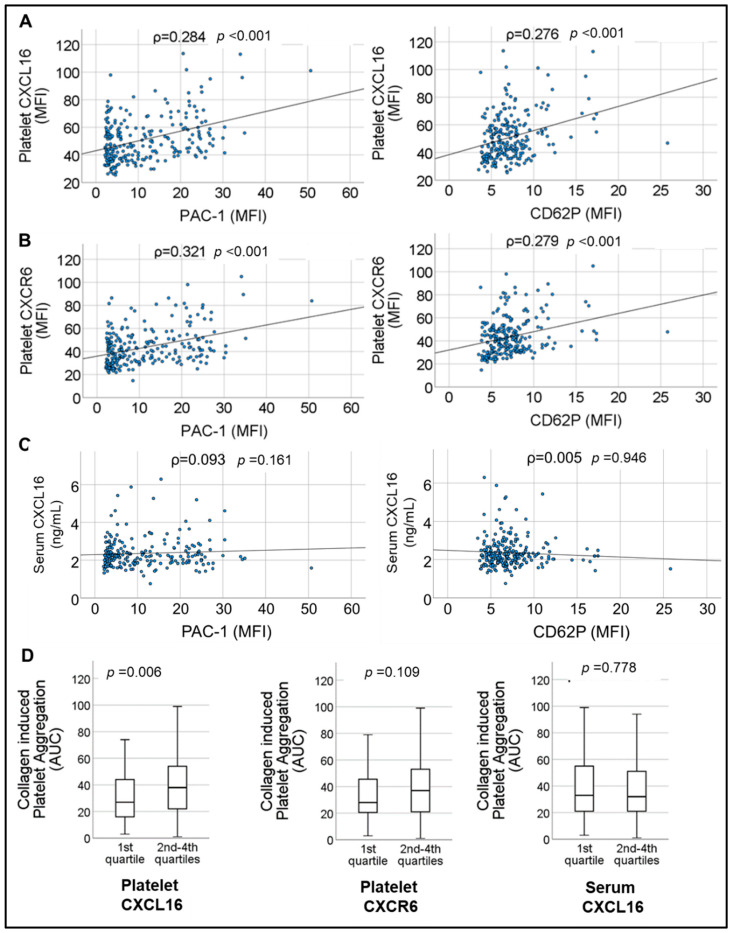
Correlations of (**A**), platelet-CXCL16 (**B**), platelet-CXCR6 and (**C**) serum levels of sCXCL16, with markers of platelet activation CD62P and PAC-1 binding in CAD patients. Correlations were assessed by Spearman’s rank correlation coefficient (ρ); *p*: level of significance; MFI: mean fluorescence intensity. (**D**). Box plots depicting collagen-induced platelet aggregation response in CAD patients corresponding to relative levels of lower (1st quartile) and higher (2nd–4th quartile) platelet-CXCL16, platelet-CXCR6, and serum levels of sCXCL16. Data represent median with 95% CI and statistical significance calculated by the Mann–Whitney U test; *p* shows the significance level.

Previously we have documented a synergistic impact of recombinant-sCXCL16 on platelet-driven thrombotic response by acting through CXCR6 in experimental studies with human (in vitro) and murine systems (arterial thrombosis model in vivo) [8]. In the current cohort of CAD patients, we observed a strong correlation between platelet-CXCL16 and platelet CXCR6 surface expression with indicators of basal platelet activation status, i.e., CD62P surface exposure denoting degranulation from α-granules (ρ = 0.284, *p* < 0.001 for platelet-CXCL16, and ρ = 0.321, *p* < 0.001 for CXCR6), and PAC-1 binding suggesting α_2b_β_3_-integrin activation (ρ = 0.276, *p* < 0.001 for platelet-CXCL16, and ρ = 0.279, *p* < 0.001 for CXCR6) (Figure 2A,B). This validated an association between the platelet-CXCL16–CXCR6 axis and thrombotic propensity in CAD. However, serum sCXCL16 did not correlate with basal circulatory platelet activation status in the absence of additional ex vivo stimulation (Figure 2C). This could be because sCXCL16 in suspension [26] only synergistically modulates platelet response to other stimuli [8]. We further explored the impact of CXCL16-CXCR6 on collagen-induced platelet aggregation ex vivo. Patients with relatively enhanced (1st quartile vs. 2nd–4th quartile) platelet-CXCL16, [median CXCL16 MFI (25th; 75th percentile) = 27.00 (16.00; 44.50) in the 1st quartile vs. 38.00 (22.00; 54.00) in the 2nd–4th quartiles] showed significantly (*p* = 0.006) increased aggregation response to collagen (Figure 2D), which was not observed with CXCR6 [median CXCR6 MFI (25th; 75th percentile) = 28.00 (20.25; 45.75) in the 1st quartile vs. 37.00 (21.00; 53.50) in the 2nd–4th quartiles, *p* = 0.109] or platelet-free serum sCXCL16 levels [median (25th; 75th percentile) = 33.00 (21.00; 55.00) in the 1st quartile of serum sCXCL16 vs. 32.00 (21.00; 51.00) in the 2nd–4th quartiles of serum sCXCL16, *p* = 0.778]. These results suggested that platelet surface association of sCXCL16 may be relevant or even mandatory for exercising a significant influence on thrombotic response. Therefore, elevated levels of circulatory sCXCL16 or CXCL16 deposited at atherosclerotic lesions and plaques [6,11,17,26] may exaggerate platelet aggregation by engaging its cognate receptor CXCR6 on the platelet surface [8].

### 2.3. Influence of Dyslipidemia and Plasma Lipids on Platelet CXCL16–CXCR6 Axis in CAD Patients

**Figure 3:**
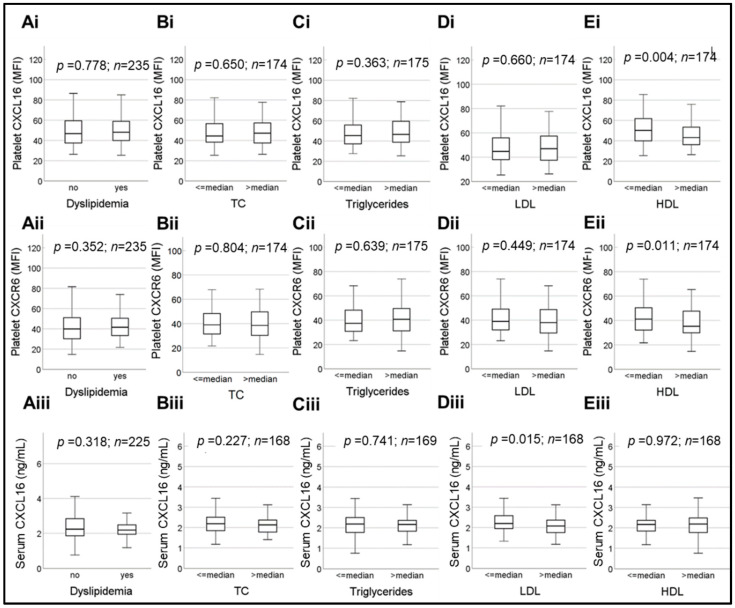
Box plots depicting changes in platelet-CXCL16, CXCR6 and serum levels of sCXCL16, with respect to (**Ai**)–(**Aiii**), dyslipidemia, relative (>median and ≤median) plasma levels of (**Bi**)–(**Biii**), total cholesterol, (**Ci**)–(**Ciii**), triglycerides, (**Di**)–(**Diii**), LDL and (**Ei**)–(**Eiii**), HDL. Data represent median with 95% CI and statistical significance calculated by Mann–Whitney U test; *p* shows the level of significance.

Although sCXCL16 is a chemokine, transmembrane-SR-PSOX/CXCL16 is a scavenger receptor for oxLDL [13,16]; the expression of this in cellular sources like endothelium [14,26], smooth muscle cells [14,15], macrophages [17] and platelets [8,9] might be influenced by circulating lipids or lipoproteins. Contrary to our expectations, in the current CAD-cohort, dyslipidemia (Figure 3A), total cholesterol (TC) (Figure 3B), and triglyceride (TG) (Figure 3C) did not have any impact on platelet-CXCL16, CXCR6 or serum sCXCL16 levels. Platelet-CXCL16 and CXCR6 surface expression were also not influenced by relative levels of plasma LDL (Figure 3D). However, HDL being associated with reduced platelet hyper-reactivity, patients with higher (>median) plasma HDL levels showed significantly (*p* = 0.004) decreased platelet-CXCL16 [median MFI (25th; 75th percentile) = 50.20 (39.80; 61.98) for HDL ≤ median and 43.15 (36.08; 53.40) for HDL > median], (Figure 3Ei) and reduced (*p* = 0.011) CXCR6 surface expression [median MFI (25th; 75th percentile) = 41.10 (32.05; 50.90) for HDL ≤ median and 35.25 (29.85; 47.75) for HDL > median] (Figure 3Eii). However, plasma HDL levels did not influence (*p* = 0.972) circulatory sCXCL16 levels [median (25th; 75th percentile) = 2.17 (1.84; 2.39) ng/mL for HDL ≤ median and 2.19 (1.76; 2.49) ng/mL for HDL > median] (Figure 3Eiii).

Transmembrane-SR-PSOX/CXCL16 being a scavenger receptor for oxLDL [6,7,8,9] and obesity being directly associated with increased risk of atherothrombosis and thromboischemic complications, we further expanded our investigation to the influence of adipokines (adiponectin, adipsin, leptin, resistin) on platelet-CXCL16–CXCR6 axis in CAD patients. None of these adipokines showed a significant association with platelet-CXCL16 or CXCR6; however, both leptin (ρ = 0.207; *p* = 0.0018) and resistin (ρ = 0.3066; *p* < 0.0001) showed a strong significant correlation with serum sCXCL16 levels and may presumably exert a pro-thrombotic influence as circulatory sCXCL16 engages platelet CXCR6.

### 2.4. Association of Platelet CXCL16–CXCR6 Axis and Cardiovascular Risk Factors in CAD

**Figure 4:**
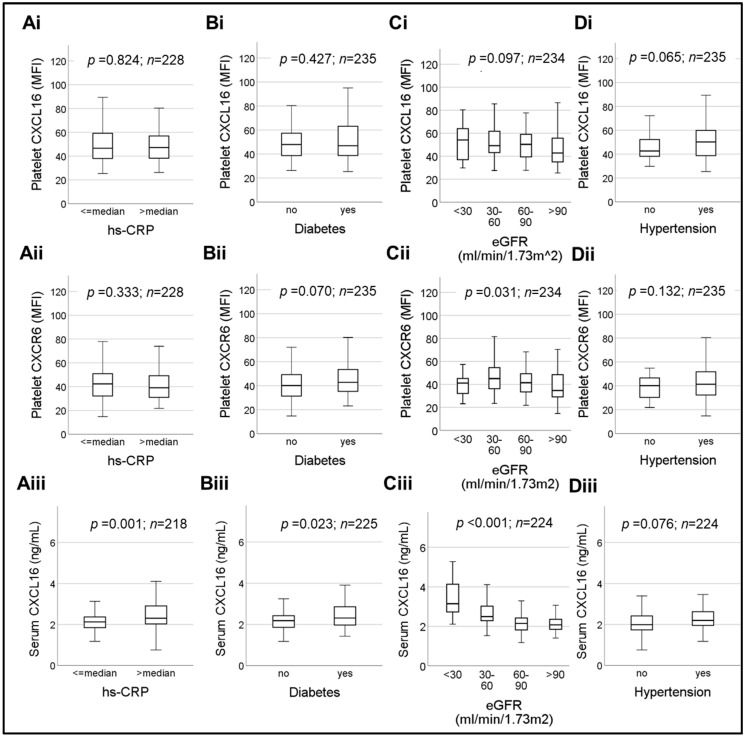
Box plots depicting changes in platelet surface-associated CXCL16, platelet-CXCR6 and serum levels of sCXCL16, with respect to (**Ai**)–(**Aiii**), relative (>median and ≤median) plasma levels of hsCRP, (**Bi**)–(**Biii**) the concurrence of diabetes, (**Ci**)–(**Ciii**), a decline in eGFR and (**Di**)- (**Diii**), concurrent presence or absence of hypertension. Data represent median with 95% CI and statistical significance calculated by Mann–Whitney U test; *p* shows the level of significance.

Next, we verified the influence of additional major cardiovascular risk factors like the inflammatory mediator hsCRP (Figure 4A), diabetes (Figure 4B), eGFR (Figure 4C) and arterial hypertension (Figure 4D) on platelet CXCL16–CXCR6 axis as compared to serum sCXCL16. Platelet-CXCL16 showed no significant difference (*p* = 0.824) between hs-CRP > median and hs-CRP ≤ median subgroups (Figure 4Ai) [median platelet-CXCL16 MFI (25th; 75th percentile) = 46.63 (37.85; 59.34) for hs-CRP ≤ median vs. 47.23 (38.19; 56.95) for hs-CRP > median]. Similar observations were made for platelet CXCR6 (*p* = 0.333) (Figure 4Aii) [median CXCR6 MFI (25th; 75th percentile) = 42.30 (32.00; 50.90) for hs-CRP ≤ median vs. 39.03 (30.94; 49.41) for hs-CRP > median] which was not influenced by hs-CRP levels. On the contrary, serum sCXCL16 levels (Figure 4Aiii) were significantly (*p* = 0.001) elevated in hs-CRP > median subgroup [median (25th; 75th percentile) = 2.31 (2.02; 2.94) ng/mL] as compared to patients with hs-CRP < median subgroup [median (25th; 75th percentile) = 2.13 (1.85; 2.37) ng/mL] suggesting a potential thrombogenic influence of hsCRP that could act through sCXCL16 engagement of platelet CXCR6 [8], as we had previously demonstrated in experimental studies.

Diabetic patients in the current cohort (Figure 4Bi–Biii) showed marginally, but significantly (*p* = 0.023), elevated levels of sCXCL16 (Figure 4Biii) [median (25th; 75th percentile) = 2.18 (1.86; 2.43) ng/mL for *w/o* diabetes vs. 2.31 (1.96; 2.88) ng/mL for w/diabetes], but exhibited no influence (*p* = 0.427) on platelet-CXCL16 (Figure 4Bi) [median MFI (25th; 75th percentile) = 47.95 (38.50; 57.38) for *w/o* diabetes vs. 46.95 (38.60; 63.24) for w/diabetes] or CXCR6 (*p* = 0.070) (Figure 4Bii) [median MFI (25th; 75th percentile) = 40.10 (34.28; 49.45) for *w/o* diabetes vs. 42.78 (34.94; 53.53) for w/diabetes].

Previous studies have shown that serum sCXCL16 concentration negatively correlates with glomerular filtrate rate (eGFR), the rate of creatinine clearance and levels of blood albumin; however, it correlates positively with proteinuria, blood urea nitrogen, and is therefore indicative of renal injury in T2DM patients [27]. Therefore, we evaluated the effect of deteriorating eGFR on the platelet CXCL16–CXCR6 axis as compared to serum sCXCL16 (Figure 4Ci–Ciii). The sCXCL16 levels in the current CAD cohort (Figure 4Ciii) increased significantly (*p* < 0.001) with gradual decline in eGFR (mL/min/1.73 m^2^) [median (25th; 75th percentile) = 3.15 (2.61; 4.25) ng/mL in **eGFR ≤ 30 mL/min/1.73 m^2^**; 2.49 (2.27; 3.05) ng/mL in **30 < eGFR ≤ 60 mL/min/1.73 m^2^**); 2.15 (1.82; 2.43) ng/mL in **60 < eGFR < 90 mL/min/1.73 m^2^**); and 2.07 (1.84; 2.37) ng/mL in **eGFR ≥ 90 mL/min/1.73 m^2^** subgroups]. Platelet surface expression of CXCR6 (Figure 4Cii) also exhibited significant (*p* = 0.031) alterations among the compared eGFR subgroups [median MFI (25th; 75th percentile) = 41.10 (31.58; 51.25) in **eGFR ≤ 30 mL/min/1.73 m^2^**, 45.05 (36.00; 54.80) in **30 <eGFR ≤ 60 mL/min/1.73 m^2^**, 41.43 (33.44; 49.35) in **60 < eGFR < 90 mL/min/1.73 m^2^**, 34.70 (28.75; 48.71) in **eGFR ≥ 90 mL/min/1.73 m^2^**]. However, platelet-CXCL16 remained unaffected [median platelet-CXCL16 MFI (25th; 75th percentile) = 54.15 (33.43; 67.40) in **eGFR ≤ 30 mL/min/1.73 m^2^**, 49.15 (42.70; 63.35) in **30 < eGFR ≤ 60 mL/min/1.73 m^2^**, 50.33 (39.33;59.11) in **60 < eGFR < 90 mL/min/1.73 m^2^**, 42.78 (34.94; 55.80) in **eGFR ≥ 90 mL/min/1.73 m^2^**] (Figure 4Ci). Confirming previous reports [27] and adding the information on platelet-CXCL16 and CXCR6, we observed a significant correlation of all three with eGFR (Table 2).

The presence or absence of hypertension did not influence platelet-CXCL16, platelet CXCR6 or serum levels of sCXCL16 (Figure 4Di–Diii). We had *n* = 195 hypertensive patients in the current cohort (Figure 4Di–Diii). Platelet-CXCL16 (Figure 4Ci) levels were median CXCL16-MFI (25th; 75th percentile) = 42.65 (37.89; 52.48) for *w/o* hypertension vs. 50.20 (38.70; 59.90) for w/hypertension. For platelet CXCR6 (Figure 4Cii) they were median CXCR6-MFI (25th; 75th percentile) = 40.13 (30.28; 47.13) for *w/o* hypertension vs. 41.30 (32.20; 51.75) for w/hypertension. Serum levels of sCXCL16 (Figure 4Biii) were median (25th; 75th percentile) = 1.99 (1.73; 2.44) ng/mL for *w/o* hypertension vs. 2.20 (1.95; 2.64) ng/mL for w/hypertension, but these parameters exhibited no significant differences (*p* > 0.05).

### 2.5. Association of Platelet CXCL16-CXCR6 with Myocardial Dysfunction and Differential Expression in CCS vs. ACS

**Figure 5:**
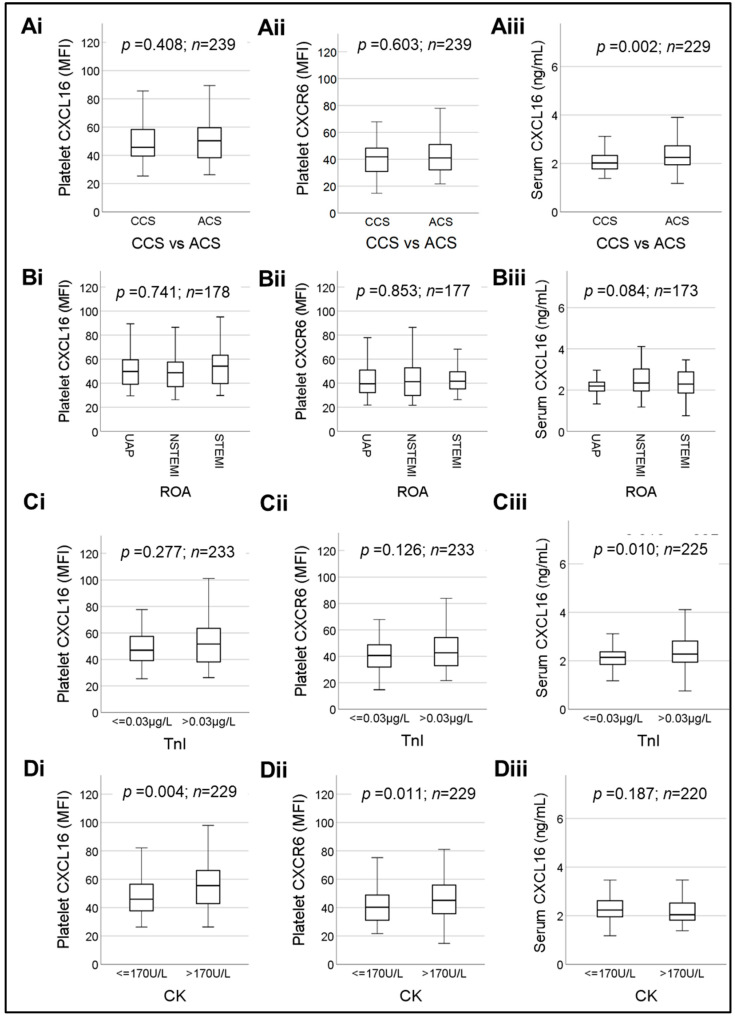
Box plots depicting changes in platelet surface-associated CXCL16, platelet-CXCR6 and serum levels of sCXCL16 in ACS vs. CCS patients (**Ai**)–(**Aiii**), with respect to the reason of admission (ROA) in ACS patients (**Bi**)–(**Biii**), with respect to troponin I (TnI) levels (**Ci**)–(**Ciii**), and creatine kinase (CK) (**Di**)–(**Diii**). Data represent median with 95% CI and statistical significance calculated by Mann–Whitney U test; *p* shows the level of significance.

Platelet-CXCL16 [median MFI (25th; 75th percentile) = 45.65 (39.10; 58.58) in CCS vs. 50.33 (38.30; 59.56) in ACS, (*p* = 0.408)] and CXCR6 surface expression [median MFI (25th; 75th percentile) = 41.80 (30.89; 48.51) in CCS vs. 41.00 (32.00; 51.00) in ACS, (*p* = 0.603)] did not alter significantly (Figure 5Ai,Aii). Contrary to this observation, serum sCXCL16 levels [median (25th; 75th percentile) = 2.02 (1.77; 2.35) ng/mL in ACS vs. 2.25 (1.94; 2.73) ng/mL in CCS] (Figure 5Aiii), were significantly (*p* = 0.002) elevated in ACS patients confirming previous reports. Since the ACS patient group is comprised of patients suffering from UAP, NSTEMI and STEMI, we performed more specific subgroup analysis. However, platelet-CXCL16 (Figure 5Bi) [median MFI (25th; 75th percentile) = 49.73 (39.10; 59.46) in UAP vs. 48.70 (36.94; 57.89) in NSTEMI vs. 54.18 (39.60; 63.83) in STEMI], platelet CXCR6 (Figure 5Bii) [median MFI (25th; 75th percentile) = 39.50 (32.09; 51.05) in UAP vs. 41.25 (29.76; 53.14) in NSTEMI vs. 41.65 (35.05; 50.20) in STEMI] and sCXCL16 (Figure 5Biii) [median (25th; 75th percentile) = 2.19 (1.95; 2.39) ng/mL in UAP vs. 2.34 (1.95; 3.03) ng/mL in NSTEMI vs. 2.29 (1.86; 2.93) ng/mL in STEMI] showed no statistically significant differences between these subgroups (Figure 5Bi–Biii).

Troponin I (TnI) and creatine kinase (CK) are sensitive biomarkers denoting myocardial necrosis that are commonly used in clinical diagnosis of ACS. Therefore, we evaluated their association with platelet CXCL16-CXCR6 and serum sCXCL16 (Figure 5C,D). The sCXCL16 levels (Figure 5Ciii) were marginally, but significantly (*p* = 0.010), elevated in patients with increased TnI [median (25th; 75th percentile) = 2.14 (1.85; 2.37) ng/mL for **TnI ≤ 0.03 μg/L** vs. 2.28 (1.93; 2.82) ng/mL for **TnI > 0.03 μg/L**]. TnI levels also showed a significant correlation with sCXCL16 levels in CAD patients (Table 2). However, patients with enhanced (>0.03 μg/L) troponin levels did not show any significant alternation in platelet-CXCL16 (*p* = 0.277) (Figure 5Ci) [median MFI (25th; 75th percentile) = 46.95 (39.10; 57.78) for **TnI ≤ 0.03 µg/L** vs. 51.63 (38.04; 63.94) for **TnI > 0.03 μg/L**] or platelet CXCR6 (*p* = 0.126) (Figure 5Cii) [median MFI (25th; 75th percentile) = 40.60 (31.89; 48.80) for **TnI ≤ 0.03 μg/L** vs. 42.65 (32.10; 54.80) for **TnI > 0.03 μg/L**].

While considering CK (Figure 5Di–Diii), we observed that platelet-CXCL16 (Figure 5Di) levels were significantly increased with elevated CK as compared to patients with CK within the normal range [median platelet-CXCL16 MFI (25th; 75th percentile) = 45.93 (37.68; 56.53) for **CK ≤ 170 U/L** vs. 55.50 (42.60; 67.30) for **CK > 170 U/L**, *p* = 0.004]. Platelet-CXCL16 levels also exhibited a significant correlation with CK in the entire cohort (Table 2). Similarly, platelet CXCR6 levels were also significantly increased (Figure 5Dii) [median CXCR6 MFI (25th; 75th percentile) = 40.23 (31.01; 48.88) for **CK ≤ 170 U/L** vs. 45.10 (35.45; 57.00) for **CK > 170 U/L**, *p* = 0.011] with increment in CK levels, although the entire data set did not correlate significantly with CK (Table 2). In contrast, sCXCL16 did not show a correlation (Table 2) or differ significantly (*p* = 0.187) with enhanced CK, as compared to the patient subgroup with normal CK [median (25th; 75th percentile) = 2.23 (1.95; 2.63) ng/mL for **CK ≤ 170 U/L** vs. 2.03 (1.81; 2.55) ng/mL for **CK > 170 U/L**], but the values did not reach statistical significance (Figure 5Diii).

### 2.6. Prognostic Association of Platelet CXCL16-CXCR6 in CAD Patients

**Figure 6:**
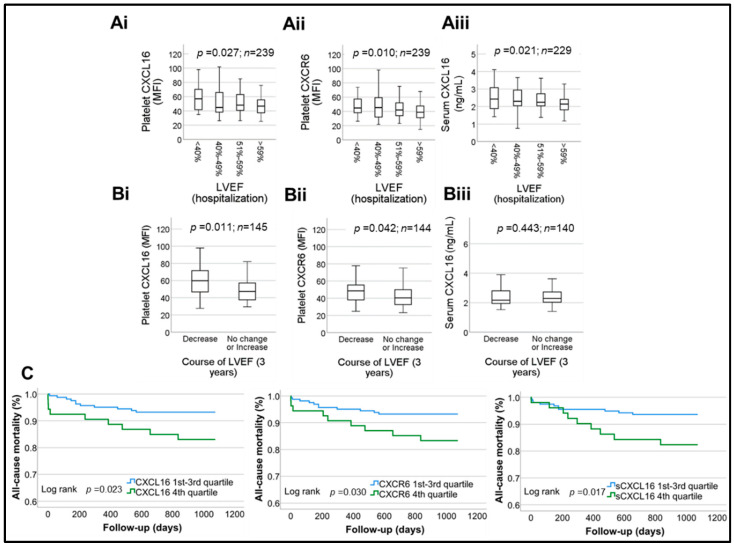
Box plots depicting changes in platelet-CXCL16, platelet-CXCR6 and serum levels of sCXCL16, with respect to deterioration in LVEF evaluated upon admission (**Ai**)–(**Aiii**), and over a follow-up period of 3 years in ACS patients (**Bi**)–(**Biii**). Data represent median with 95% CI and statistical significance calculated by Mann–Whitney U test; *p* shows the level of significance. (**C**). Kaplan–Meier curves illustrating the likelihood of all cause of mortality stratified according to platelet-CXCL16 MFI quartile subgroups, platelet-CXCR6 MFI quartile subgroups, serum levels of sCXCL16 quartile subgroups. *p*: level of significance.

Since we observed an association between platelet-CXCL16, platelet CXCR6 and CK levels, we validated their association with LVEF in patients (Table 3). Higher levels of both platelet-CXCL16 and CXCR6 corresponded with a decline in LVEF measured at admission among CAD patients. For platelet-CXCL16 (Figure 6Ai) the values were median platelet-CXCL16 MFI (25th; 75th percentile) = 56.95 (41.65; 70.75) in **LVEF < 40%** vs. 44.83 (38.23; 66.21) in **40% ≤ LVEF ≤ 49%** vs. 48.10 (39.70; 63.50) in **49% < LVEF ≤ 59%** vs. 46.70 (37.08; 55.55) in **LVEF > 59%,** *p* = 0.027. For platelet-CXCR6 (Figure 6Aii) the values were median CXCR6 MFI (25th; 75th percentile) = 44.80 (37.05; 62.55) in **LVEF < 40%** vs. 45.30 (31.94; 60.50) in **40% ≤ LVEF ≤ 49%** vs. 41.98 (33.46; 52.46) in **49% < LVEF ≤ 59%** vs. 38.85 (31.19; 47.70) in **LVEF > 59%,** *p* = 0.010. Serum sCXCL16 levels (Figure 6Aiii) corresponded modestly (*p* = 0.021) with deterioration in LVEF [median (25th; 75th percentile) = 2.43 (1.88; 3.10) ng/mL in **LVEF < 40%** vs. 2.30 (2.03; 3.01) ng/mL in **40% ≤ LVEF ≤ 49%** vs. 2.25 (2.01; 2.74) ng/mL in **49% < LVEF ≤ 59%** vs. 2.14 (1.81; 2.41) ng/mL in **LVEF > 59%**]. Accordingly, all three parameters showed inverse correlation with baseline LVEF% (Table 2.)

We could evaluate the deterioration course of LVEF in ACS patients after a follow-up period of 3 years (Figure 6Bi–Biii). Baseline platelet-CXCL16 values significantly (*p* = 0.011) corresponded with the course of LVEF deterioration (Figure 6Bi) over 3 years [median platelet-CXCL16 MFI (25th; 75th percentile) = 56.50 (40.00; 71.95) in the **LVEF decreased** group vs. 47.00 (38.50; 57.28) in the **stable LVEF** group]. Similarly, higher platelet-CXCR6 evaluated upon admission significantly (*p* = 0.042) corresponded with the course of LVEF deterioration over 3 years [median CXCR6 MFI (25th; 75th percentile) = 48.00 (32.40; 58.78) in **LVEF decreased** group vs. 40.63 (32.23; 49.45) in the group with **stable LVEF**] (Figure 6Bii). However, we did not observe a direct correlation between platelet-CXCL16, platelet-CXCR6 measured at baseline, and LVEF% monitored after 3 years (Table 2). No significant differences (*p* = 0.443) or correlations (Table 2) were observed in the compared LVEF-follow-up groups [median (25th; 75th percentile) = 2.15 (1.95; 2.49) in **LVEF decreased** group vs. 2.24 (1.91; 2.64) in **stable LVEF** group] with respect to serum sCXCL16 levels (Figure 6Biii).

Furthermore, we ascertained the prognostic relevance of platelet CXCL16–CXCR6 axis as compared to serum sCXCL16 levels on all-cause death in CAD patients (*n* = 217). Higher baseline expression of platelet-CXCL16, platelet-CXCR6, and serum sCXCL16 were all associated with poor prognosis of CAD patients (Figure 6C), as seen in the Kaplan–Maier curves. Baseline characteristics of the complete study cohort stratified according to prognosis are presented in Table 3.

As shown in Cox PH regression analysis in Table 4, platelet-CXCL16 (*p* = 0.039) was validated as an independent risk factor in influencing prognosis for all-cause of mortality. Initially, we performed a multivariable analysis of the covariables with age and LG troponin I remained independently associated with all-cause mortality. A further variable selection step was performed for the three CXC (serum sCXCL16, platelet-CXCL16, platelet-CXCR6) chemokine-chemokine receptor variables, keeping age and LG troponin I in the analysis. The only variable significant was platelet CXCL16. However, the power of the study was not large enough to establish that platelet CXCL16 is superior to CXCR6.

## 3. Discussion

**Figure 7:**
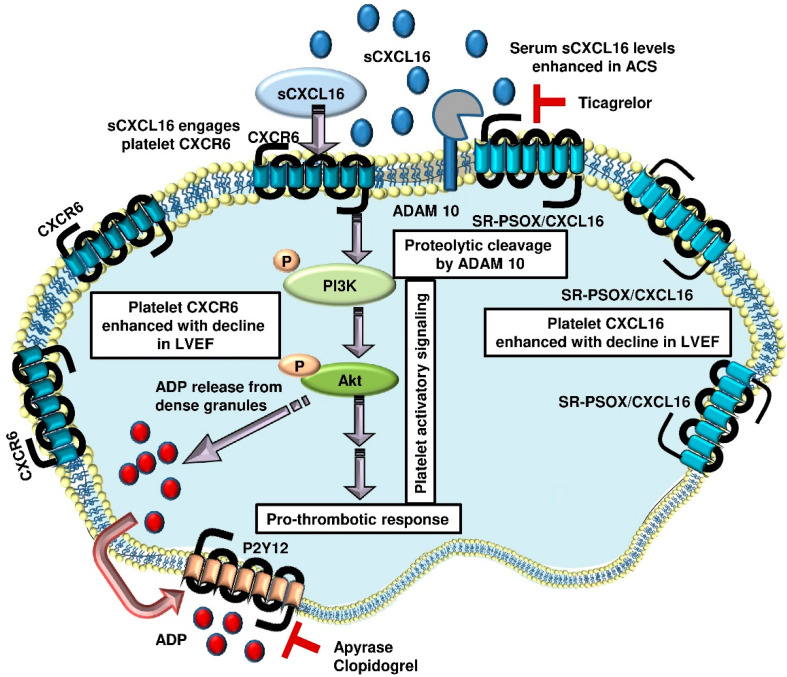
Schematic diagram showing that platelet surface associated CXCL16 levels are enhanced in CAD patients with a decline in LVEF, and so is the surface expression of CXCR6. Proteolytic cleavage of transmembrane-SR-PSOX/CXCL16 from activated platelets generates circulatory sCXCL16, levels of which are also elevated in CAD patients. This effect is apparently counteracted following ticagrelor administration. Circulatory sCXCL16 may engage CXCR6 on the platelet surface and trigger the activatory signaling cascade involving PI3K-Akt, leading to dense granule release of ADP and a profound pro-thrombotic drive. ADP engagement of P2Y_12_ and a subsequent positive feedback loop is therefore counteracted by ADP degrading apyrase and P2Y_12_ antagonist clopidogrel, suggesting the clinical significance of platelet CXCL16–CXCR6 axis.

The current clinical investigation was undertaken to explore the pathophysiological significance of the platelet-CXCL16–CXCR6 axis [8] in influencing thrombotic disposition and its prognostic association in CAD patients (Figure 7). This investigation might be considered a translational extension of our previous experimental study, which documented a synergistic impact of recombinant-sCXCL16 on platelet-driven thrombotic responses that are executed through CXCR6 [8] and delineated the molecular mediators downstream. We validated these observations in experiments with human platelets (in vitro) and a murine model of arterial thrombosis in vivo. The currently observed correlation between platelet-CXCL16 and CXCR6 surface expression with markers of platelet activation validates our experimental finding that ligation of CXCR6 by sCXCL16 may support a pro-thrombotic mode of action in pathophysiological settings like CAD. Since serum levels of free sCXCL16 did not correlate with platelet activation or aggregatory potential, the surface association of sCXCL16 on platelets seems critical in mediating the pro-thrombotic effects through CXCR6 ligation. In potential pathological settings, elevated circulatory sCXCL16 or CXCL16 deposited at atherosclerotic lesions and plaques [6,7,28,29] may exaggerate platelet aggregation by engaging platelet CXCR6 [28].

A significant correlation between platelet-CXCL16 and CXCR6 and a negative correlation between platelet count and platelet-free serum levels of sCXCL16 as currently observed also suggests that platelets may sequester circulatory sCXCL16, levels of which are elevated in ACS patients [23,28]. Platelets in their activated state may also shed transmembrane-SR-PSOX/CXCL16, thereby adding to the circulatory levels of sCXCL16 derived from other vascular cells and leukocytes. We compared the levels of platelet surface-associated CXCL16, surface-expressed CXCR6 and serum sCXCL16 with respect to treatment with P2Y_12_ antagonists clopidogrel and ticagrelor. Although none of the P2Y_12_ antagonists showed any impact on either platelet-CXCL16 or CXCR6, ticagrelor significantly reduced serum sCXCL16 levels, which might be attributed to the counteraction of transmembrane-SR-PSOX/CXCL16 shedding from activated platelets during blood clotting. It is worth considering that the pro-thrombotic effects of sCXCL16 mediated through platelet CXCR6 are antagonized by ADP degrading apyrase and ADP receptor P2Y_12_ antagonists [8]. The currently observed effects of ticagrelor add to the potential benefits of P2Y_12_ antagonists in reducing pro-thrombotic sCXCL16 levels in CAD patients.

Contrary to our expectations, platelet-CXCL16, CXCR6 or serum sCXCL16 levels were not influenced by dyslipidemia to a significant extent or even by individual lipid (total cholesterol, triglyceride) or lipoprotein (LDL) profiles, all of which are major risk factors for ACS [30]. On the other hand, as HDL exerts some regulatory impact on platelet activation and aggregation [31,32], higher plasma HDL levels corresponded with significantly reduced platelet-CXCL16 and CXCR6 surface expression. Among other cardiovascular risk factors, only sCXCL16 levels were affected considerably by inflammatory mediator hsCRP, the concurrence of diabetes, and a decline in eGFR. Hypertension seemingly did not affect platelet-CXCL16, serum sCXCL16 or CXCR6 surface expression on platelets.

Platelet-CXCL16 and CXCR6 did not alter significantly between CCS and ACS patients. However, our data confirmed previous findings that serum sCXCL16 levels are significantly enhanced in ACS patients [23]. Both TnI and CK are indicators of cardiac injury. Platelet-CXCL16 and CXCR6 did not show any association with TnI, but higher levels of both corresponded with increased CK. Elevated serum sCXCL16 levels in the current cohort corresponded with peak TnI levels in accordance with previous reports [19,23,28]. Platelet-CXCL16 and CXCR6 levels were relatively higher in patients with worsened LVEF ascertained during the hospital stay and a follow-up measurement after three years, suggesting its potential association with myocardial dysfunction. Kaplan–Meier analyses of data from the current cohort showed a significant association between platelet-CXCL16, platelet CXCR6, sCXCL16, and all-cause of mortality in the three years of the follow-up period, but not explicitly for MI. We further analyzed the differences in clinical and analytical/test variants between alive and deceased patients in our cohort. Based on multivariable analyses after adjusting for clinical variables, i.e., age, NYHA class, LVEF% upon admission, hs-CRP and Troponin I, which were significantly different between alive and deceased patients, only platelet-CXCL16 remained as an independent prognostic indicator for all causes of mortality in the current cohort. Our investigation does not undermine the effectiveness of sCXCL16 as a predictive marker of MI, but it puts forth the association between platelet-CXCL16 and prognosis for all-cause of mortality in CAD patients. In the subgroup analysis of the 5142 patients randomized in the PLATO trial [22], clinical outcome was specifically ascertained in ACS patients as compared to CAD (including CCS and ACS) patients in our cohort. The PLATO investigators considered sudden MI or stroke and composite of cardiovascular death as clinical endpoints. The investigators had also concluded that serum CXCL16 measured upon admission was independently associated with cardiovascular death but not explicitly with ischemic events [22]. Investigators of the HUNT2 cohort in Norway estimated the prognostic effectiveness of sCXCL16 as a potential risk factor for MI in a large population of 58,761, who were followed for the first incidence of MI over a period of 11.3 years, and registered 1587 incidents. Comparing sCXCL16 values in MI patients to 3959 age- and sex-matched controls, they observed only subtle differences in sCXCL16 levels; nevertheless, there was a substantially increased risk of MI amongst those in the highest quartile [23].

***Conclusions:*** The major findings of the present study are that: **(i)** Platelet surface-associated CXCL16 and CXCR6 surface expression are significantly associated with activation status (α-granule degranulation and α_2b_β_3_-integrin activation) of platelets in circulation; **(ii)** Higher platelet-CXCL16 and CXCR6 surface expression also corresponded with a higher degree of activation potential in platelets ascertained by collagen-induced platelet aggregation response ex vivo. Engagement of circulatory sCXCL16 to platelet surface expressed CXCR6 is seemingly essential to trigger the activatory signaling cascade to drive platelet activation [8]. Therefore, platelet-free serum levels of sCXCL16 did not correlate with platelet activation markers or aggregatory potential; **(iii)** Although platelet-CXCL16, CXCR6 expression did neither change significantly between ACS and CCS patients, nor correlated significantly with Tn I levels, increased platelet-CXCL16 and CXCR6 corresponded with higher CK and progressively deteriorated LVEF; **(iv)** Consequently, elevated baseline platelet-CXCL16 and CXCR6 were significantly associated with time to all-cause of mortality/death (Kaplan–Meier curve); **(v)** In multivariable regression analysis, platelet-CXCL16 was found to be independently associated with time to all-cause of mortality.

***Study limitations:*** The current investigation has several limitations: **(i)** We could measure the laboratory parameters, i.e., platelet-CXCL16, CXCR6 and sCXCL16, only upon admission, which leaves potential alterations in these parameters over time (3 years of follow-up period) and the impact of subsequent anti-platelet therapy to speculation. We could, however, evaluate LVEF in ACS patients after the follow-up period; **(ii)** 23 out of 240 patients (9.6%) in the overall CAD cohort were lost to follow-up; **(iii)** The size of the current cohort was relatively small (*n* = 240), being a single-center study, as compared to a large number of patients enrolled in the PLATO subgroup analysis [22] and HUNT2 cohort [23]; **(iv)** A relative comparison of platelet-CXCL16, platelet CXCR6 and serum sCXCL16 levels in an age-matched non-CAD control group is currently lacking; **(v)** We did not offer any mechanistic basis behind the observations from the clinical cohort, but have previously reported the molecular mediators in the platelet CXCL16–CXCR6 axis that can regulate thrombotic response [8,9]. Nevertheless, the results from this cohort present a novel translational aspect on the relevance of the platelet CXCL16–CXCR6 axis in influencing thrombotic propensity and prognosis in CAD patients; this will encourage more extensive clinical investigations in the future.

## 4. Materials and Methods

**Materials:** Mouse anti-human CXCL16-PE, mouse anti-human CXCR6-PE, and human CXCL16 Quantikine ELISA kit were procured from R&D systems; anti-human CD62P-FITC and CD42b-FITC were from Beckman Coulter; anti-human CD42b-PE, PAC-1-FITC were procured from BD Biosciences. Reagents for platelet aggregation tests in the Multiplate^®^ analyzer were procured from Roche Diagnostics. Human metabolic panel 1 Legendplex^®^ cytometric bead array kit for adipokines was from Biolegend.

**Study subjects:** Platelet surface-associated CXCL16 (transmembrane-SR-PSOX/CXCL16 + surface-bound sCXCL16) and CXCR6 were evaluated in *n* = 240 consecutive patients with symptomatic CAD. Blood samples were collected during PCI, and 20 mL of blood was drawn through the catheter sheath into a sterile 20 mL syringe from all participants. Immediately, collected blood was put into tubes containing the following anticoagulants: citrate-phosphate-dextrose solution with adenine (CPDA) (for flow cytometric analysis), hirudin (for impedance aggregometry). Furthermore, blood was filled into specific tubes without anticoagulants to collect serum. Chronic coronary syndrome-(CCS) and ACS were defined as elaborated previously [25,33,34]. Patients admitted to the Department of Cardiology and Angiology, University Hospital Tübingen, gave written informed consent. The study was approved by the institutional ethics committee (270/2011BO1, 237/2018BO2) and complied with the declaration of Helsinki and good clinical practice guidelines. Baseline characteristics of the patient cohort are given in Table 1 and Table 4.

**Surface expression of SR-PSOX/CXCL16 and CXCR6 on platelets:** Platelets in whole blood samples were analyzed for platelet surface-associated CXCL16 and CXCR6, gating for the platelet-specific marker GPIb (CD42b). Blood collected in CPDA was diluted at 1:50 with PBS (Gibco) and incubated with the respective fluorochrome-conjugated antibodies—mouse monoclonal anti-human SR-PSOX/CXCL16-PE or mouse monoclonal anti-human CXCR6-PE, and mouse anti-human GPIb (CD42b)-FITC for 30 min at room temperature. After staining, the samples were fixed with 0.5% paraformaldehyde and analyzed by flow cytometry (FACS-Calibur flow cytometer BD Biosciences) [8,9,34].

**Platelet activation markers CD62P and PAC-1 binding:** Platelet activation markers CD62P surface expression (for degranulation from α-granules) and PAC-1 binding (α_2b_β_3_-integrin activation) at basal state without *ex vivo* stimulation were also checked by whole blood flow cytometry gating for platelet specific marker GPIb (CD42b). CPDA anticoagulated blood from CAD patients (*n* = 240) was diluted at 1:50 with PBS (Gibco) and incubated with anti-human CD62P-FITC, or PAC-1-FITC, and mouse anti-human GPIb (CD42b)-PE for 30 min at room temperature. Samples were fixed with 0.5% paraformaldehyde and analyzed by flow cytometry [34].

**Serum levels of sCXCL16:** Serum levels of CXCL16 [28] were estimated in biobank serum samples from CAD patients using the Quantikine-ELISA kit according to the manufacturer’s instructions.

**Serum levels of adipokines:** Serum levels of adipokines (adiponectin, adipsin, leptin, resistin) were estimated in biobank serum samples from CAD patients using Legendplex^®^—Human Metabolic panel-1 as per manufacturer’s instructions.

**Follow-up study to assess prognostic impact:**

***LVEF:*** A parameter that can be measured relatively easily and gives good information about myocardial function is LVEF%. However, detailed echocardiography is sometimes impossible in an emergency like ACS. Therefore, the intra-hospital course of LVEF% was evaluated in CAD patients using transthoracic echocardiography. After a median of 3 years, we re-evaluated LVEF% in ACS patients. Two-dimensional echo LVEF was assessed using Simpson’s biplane method of discs by manual planimetry of the endocardial border in end-diastolic and end-systolic frames.

***Clinical endpoints:*** All patients enrolled in the study were followed up for all-cause of mortality for 1080 days after study inclusion. Follow-up was performed by telephonic interview and/or review of patients’ charts on readmission by investigators blinded to laboratory results [35]. A total of 23 out of 240 patients (9.6%) were lost to follow-up.

**Statistical Analysis:** Statistical analyses were performed using SPSS version 26.0 (SPSS Inc., Chicago, IL, USA). Chi-square tests, Student’s *t*-test and one-way Cox analyses were applied as appropriate to analyze baseline characteristics. Non-parametric data, including mean fluorescence intensity (MFIs), are presented as median values (25th; 75th percentile) in the form of box plots and compared using the Mann–Whitney U test. Correlations were assessed using Spearman’s rank correlation coefficient (ρ) and are presented as scatter plots. The level of significance was determined as *p* < 0.05. Cox proportional hazard (PH) regression with forward variable selection (Inclusion *p* = 0.05, exclusion *p* = 0.10) was applied to investigate associations between survival endpoint and platelet-CXCL16, sCXCL16 as well as CXCR6. The time-dependent covariate method was used to check the proportional hazard assumption of the Cox model. Survival functions were estimated by Kaplan–Meier curves. The log-rank test was applied to compare survival functions between high vs. low platelet-CXCL16, sCXCL16 as well as CXCR6 levels. All statistical tests were two-tailed, and the statistical significance level was defined as *p* < 0.05 [35].

## Figures and Tables

**Table 1:** Baseline characteristics of the overall cohort of CAD patients (*n* = 240), broadly categorized according to ACS and CCS.

	CCS (*n* = 62)	ACS (*n* = 178)	*p* Value
**Age, years (mean ± SD)**	70 (±10.0)	70 (±11.1)	0.942
**Male, *n* (%)**	50 (80.6%)	141 (79.2%)	0.810
**NYHA I (%)**	21 (34.4%)	31 (17.4%)	**<0.001**
**NYHA II (%)**	31 (50.0%)	78 (43.8%)	
**NYHA III (%)**	10 (16.1%)	36 (20.2%)	
**NYHA IV (%)**	0 (0.0%)	33 (18.5%)	
**CCS I (%)**	33 (53.2%)	26 (14.6%)	**<0.001**
**CCS II (%)**	12 (19.4%)	23 (12.9%)	
**CCS III (%)**	17 (27.4%)	47 (26.4%)	
**CCS IV (%)**	0 (0.0%)	82 (46.1%)	
**Coronary sclerosis (%)**	6 (9.7%)	9 (5.1%)	0.349
**1-vessel disease (%)**	8 (12.9%)	35 (19.7%)	
**2-vessel disease (%)**	17 (27.4%)	40 (22.5%)	
**3-vessel disease (%)**	31 (50.0%)	94 (52.8%)	
**Left main disease (%)**	21 (33.9%)	40 (22.5%)	0.076
**No to minimal MVR (%)**	13 (21.0%)	42 (5.0%)	0.364
**Mild MVR (%)**	42 (67.7%)	110 (23.6%)	
**Moderate MVR (%)**	7 (11.3%)	20 (11.2%)	
**Severe MVR (%)**	0 (0.0%)	6 (3.4%)	
**ICD (%)**	3 (4.8%)	3 (1.7%)	0.171
**Recent MI (%)**	5 (8.1%)	4 (2.2%)	**0.038**
**CXCL16 MFI** **(25th–75th percentile)**	45.7 (39.1–58.6)	50.3 (38.3–59.6)	0.408
**CXCR6 MFI** **(25th–75th percentile)**	41.8 (30.9–48.5)	41.0 (32.0–51.0)	0.603
**sCXCL16 ng/mL** **(25th–75th percentile)**	2.0 (1.8–2.3)	2.2 (1.9–2.7)	**0.002**
**Cardiovascular risk factors, *n* (%)**			
**Arterial hypertension**	50 (80.6%)	145 (81.5%)	0.893
**Dyslipidemia**	44 (71.0%)	95 (53.4%)	**0.015**
**Diabetes mellitus**	19 (30.6%)	47 (26.4%)	0.520
**Current smokers**	22 (35.5%)	47(26.4%)	0.173
**Obesity**	11 (17.7%)	23 (12.9%)	0.349
**Family history**	15 (24.2%)	37 (20.8%)	0.576
**Echocardiography**			
**LVEF, baseline%** **(mean ± SD)**	53 (±9.7)	52 (±10.7)	0.443
**LVEF, Follow-up%** **(mean ± SD)**	53 (±9.8)	51 (±10.5)	0.445
**Laboratory assessment upon admission, median (25th percentile–75th percentile)**			
**Platelets, x 1000/µL**	211.0 (182.0–258.8)	210.0 (173.5–255.0)	0.559
**eGFR, mL/m²**	81.8 (67.9–94.0)	74.4 (61.0–92.5)	0.284
**hs-CRP, mg/dL (LG)**	−0.88 (−1.40–−0.32)	−0.39 (−1.05–0.07)	**0.002**
**Troponin I, ng/dL (LG)**	−1.52 (−1.52–−1.52)	−1.52 (−1.52–−0.39)	**<0.001**
**NT pro-BNP, ng/L**	99 (21–387)	393 (162–2696)	0.088
**CK, U/L (LG)**	2.03 (1.79–2.15)	2.00 (1.85–2.26)	0.150
**HDL, mg/dL**	49 (39–56)	45 (36–59)	0.345
**LDL, mg/dL**	95.5 (65.5–127.5)	106.0 (83.0–133.0)	0.057
**TC, mg/dL**	166.5 (127.5–192.5)	167.0 (146.0–201.0)	0.337
**Triglycerides, mg/dL**	124.0 (93.8–161.3)	128.0 (87.0–181.0)	0.776
**Medication at admission, *n* (%)**			
**ASA**	31 (50.0%)	75 (42.1%)	0.515
**Clopidogrel**	15 (23.1%)	19 (10.7%)	**0.016**
**Prasugrel**	2 (3.2%)	3 (1.7%)	0.521
**Ticagrelor**	7 (11.3%)	8 (4.5%)	0.081
**DAPT**	19 (30.6%)	25 (14.0%)	**0.007**
**ACE inhibitors**	23 (37.1%)	51 (28.7%)	**0.359**
**ARB**	19 (30.6%)	44 (24.7%)	0.543
**Aldosterone inhibitors**	5 (8.1%)	9 (5.1%)	0.467
**Thiazide diuretics**	10 (16.1%)	24 (13.5%)	0.784
**Loop diuretics**	8 (12.9%)	21 (11.8%)	0.992
**Calcium channel blockers**	13 (21.0%)	22 (12.4%)	0.086
**Beta-blockers**	35 (56.5%)	76 (42.7%)	0.127
**Statins**	37 (59.7%)	70 (39.3%)	**0.011**
**Oral anticoagulants**	12 (19.4%)	23 (12.9%)	0.281
**Medication at discharge, *n* (%)**			
**ASA**	52 (83.9%)	145 (81.5%)	0.598
**Clopidogrel**	29 (46.8%)	56 (31.5%)	**0.027**
**Prasugrel**	5 (8.1%)	30 (16.9%)	0.092
**Ticagrelor**	13 (21.0%)	55 (30.9%)	0.137
**DAPT**	43 (69.4%)	122 (68.5%)	0.866
**ACE inhibitors**	27 (43.5%)	88 (49.4%)	0.433
**ARB**	20 (32.3%)	60 (33.7%)	0.850
**Aldosterone inhibitors**	7 (11.3%)	42 (23.6%)	**0.039**
**Thiazide diuretics**	11 (17.7%)	32 (18.0%)	0.978
**Loop diuretics**	11 (17.7%)	31 (17.4%)	0.943
**Calcium channel blockers**	19 (30.6%)	44 (24.7%)	0.349
**Beta-blockers**	39 (62.9%)	128 (71.9%)	0.183
**Statins**	55 (88.7%)	156 (87.6%)	0.733
**Oral anticoagulants**	11 (17.7%)	32 (18.0%)	0.978
**PCI vs. CABG vs. conservative treatment, *n* (%)**			
**PCI**	42 (67.7%)	143 (80.3%)	
**CABG**	1 (1.6%)	3 (1.7%)	
**Conservative treatment**	19 (30.6%)	32 (18.0%)	0.110

NYHA = New York Heart Association; CCS (I–IV) = Canadian Cardiovascular Society; LG: Logarithm calculated for base 10.

**Table 2:** Correlations of serum sCXCL16, platelet-CXCL16, and platelet surface-expressed CXCR6 with clinical parameters and platelet aggregation in CAD patients. Spearman correlation coefficients with respective *p*-values are tabulated below.

Diagnostic Parameters	Platelet CXCL16	Platelet CXCR6	sCXCL16
**Collagen-induced platelet aggregation**	ρ = 0.167 ***p* = 0.014**	ρ = 0.153 ***p* = 0.025**	ρ = −0.055 *p* = 0.429
**Total cholesterol**	ρ = −0.015 *p* = 0.849	ρ = −0.078 *p* = 0.338	ρ = −0.136 *p* = 0.098
**Triglycerides**	ρ = 0.023 *p* = 0.773	ρ = −0.053 *p* = 0.512	ρ = −0.011 *p* = 0.896
**LDL**	ρ = −0.021 *p* = 0.799	ρ = −0.070 *p* = 0.387	ρ = −0.089 *p* = 0.283
**HDL**	ρ = −0.121 *p* = 0.136	ρ = −0.104 *p* = 0.197	ρ = −0.016 *p* = 0.844
**Hs-CRP**	ρ = 0.008 *p* = 0.928	ρ = −0.022 *p* = 0.749	ρ = 0.226 ***p* = 0.001**
**eGFR**	ρ = −0.160 ***p* = 0.020**	ρ = −0.209 ***p* = 0.002**	ρ = −0.406 ***p* < 0.001**
**TnI**	ρ = 0.085 *p* = 0.218	ρ = 0.065 *p* = 0.343	ρ = 0.155 ***p* = 0.027**
**CK**	ρ = 0.176 ***p* = 0.011**	ρ = 0.108 *p* = 0.118	ρ = −0.105 *p* = 0.141
**LVEF% baseline**	ρ = −0.197 ***p* = 0.004**	ρ = −0.224 ***p* < 0.001**	ρ = −0.186 ***p* = 0.007**
**Course of LVEF%**	ρ = −0.008 *p* = 0.913	ρ = −0.035 *p* = 0.645	ρ = −0.003 *p* = 0.965

**Table 3:** Patient characteristics categorized with respect to prognosis.

	Patients Alive (*n* = 197)	Patients Deceased (*n* = 20)	*p* Value (Cox)
**Age, years (mean ± SD)**	70 (±10.5)	78 (±7.6)	**0.002**
**Male, *n* (%)**	154 (78.2%)	18 (90.0%)	0.245
**ACS, *n* (%)**	142 (72.1%)	17 (85.0%)	0.227
**NYHA I (%)**	41 (20.8%)	3 (15.0%)	**0.004**
**NYHA II (%)**	97 (49.2%)	6 (30.0%)	
**NYHA III (%)**	39 (19.8%)	3 (15.0%)	
**NYHA IV (%)**	20 (10.2%)	8 (40.0%)	
**CCS I (%)**	53 (26.9%)	5 (25.0%)	0.184
**CCS II (%)**	29 (14.7%)	1 (5.0%)	
**CCS III (%)**	51 (25.9%)	3 (15.0%)	
**CCS IV (%)**	64 (32.5%)	11 (55.0%)	
**Coronary sclerosis (%)**	13 (6.6%)	0 (0.0%)	**0.033**
**1-vessel disease (%)**	37 (18.8%)	1 (5.0%)	
**2-vessel disease (%)**	46 (23.4%)	4 (20.0%)	
**3-vessel disease (%)**	101 (51.3%)	15 (75.0%)	
**Left main disease (%)**	49 (24.9%)	8 (40.0%)	0.142
**No to minimal MVR (%)**	45 (22.8%)	1 (5.0%)	0.103
**Mild MVR (%)**	124 (62.9%)	13 (65.0%)	
**Moderate MVR (%)**	22 (11.2%)	4 (20.0%)	
**Severe MVR (%)**	5 (2.5%)	1 (5.0%)	
**ICD (%)**	5 (2.5%)	1 (5.0%)	0.473
**Recent MI (%)**	7 (3.6%)	1 (5.0%)	0.756
**CXCL16 MFI** **(25th–75th percentile)**	47.1 (38.0–58.7)	53.4 (40.1–71.1)	0.102
**CXCR6 MFI** **(25th–75th percentile)**	40.8 (31.7–49.9)	48.8 (40.9–65.9)	**0.040**
**sCXCL16 ng/mL** **(25th–75th percentile)**	2.2 (1.9–2.5)	2.4 (2.1–3.4)	**0.005**
**CXCL16** **(4th quartile vs. rest)**	44 (22.3%)	9 (45.0%)	**0.029**
**CXCR6** **(4th quartile vs. rest)**	45 (22.8%)	9 (45.0%)	**0.036**
**sCXCL16** **(4th quartile vs. rest)**	42 (21.3%)	9 (45.0%)	**0.022**
**Cardiovascular risk** **factors, *n* (%)**			
**Arterial hypertension**	163 (82.7%)	17 (85.0%)	0.928
**Dyslipidemia**	118 (59.9%)	8 (40.0%)	0.072
**Diabetes mellitus**	49 (24.9%)	9 (45.0%)	0.068
**Current smokers**	58 (29.4%)	3(15.0%)	0.177
**Obesity**	28 (14.2%)	2 (10.0%)	0.592
**Family history**	46 (23.4%)	2 (10.0%)	0.191
**Echocardiography**			
**LVEF, baseline% ** **(mean ± SD)**	53 (±9.9)	43.3 (±13.6)	**<0.001**
**LVEF, Follow-up% ** **(mean ± SD)**	53 (±9.9)	42.9 (±12.4)	**0.001**
**Laboratory assessment upon admission,** **median (25th percentile–75th percentile)**			
**Platelets, ×1000/µL**	212.0 (176.0–255.0)	182.0 (131.8–230.5)	0.765
**eGFR, mL/m²**	76.2 (63.2–93.0)	60.1 (25.6–93.3)	0.012
**hs-CRP, mg/dL (LG)**	−0.61 (−1.19–−0.04)	−0.13 (−0.74–0.73)	**0.010**
**Troponin I, ng/dL (LG)**	−1.52 (−1.52–−1.02)	−0.74 (−1.52–0.70)	**0.004**
**NT pro-BNP, ng/L**	354 (99–2012)	393 (183–3379)	0.921
**CK, U/L (LG)**	1.99 (1.82–2.23)	1.90 (1.72–2.97)	0.068
**HDL, mg/dL**	47 (39–58)	39 (28–55)	0.154
**LDL, mg/dL**	103.5 (77.3–132.8)	86.0 (84.0–110.0)	0.644
**TC, mg/dL**	167.0 (145.5–195.5)	165.0 (141.0–179.0)	0.443
**Triglycerides, mg/dL**	125.0 (88.3–167.8)	142.0 (95.5–288.0)	0.141
**Medication at admission, *n* (%)**			
**ASA**	89 (45.2%)	7 (35.0%)	0.435
**Clopidogrel**	28 (14.2%)	2 (10.0%)	0.748
**Prasugrel**	4 (2.0%)	1 (5.0%)	0.334
**Ticagrelor**	11 (5.6%)	2 (10.0%)	0.377
**DAPT**	34 (17.3%)	4 (20.0%)	0.591
**ACE inhibitors**	58 (29.4%)	7 (35.0%)	0.333
**ARB**	54 (27.4%)	3 (15.0%)	0.317
**Aldosterone inhibitors**	12 (6.1%)	1 (5.0%)	0.944
**Thiazide diuretics**	30 (15.2%)	2 (10.0%)	0.671
**Loop diuretics**	23 (11.7%)	4 (20.0%)	0.208
**Calcium channel blockers**	29 (14.7%)	1 (5.0%)	0.297
**Beta-blockers**	95 (48.2%)	8 (40.0%)	0.760
**Statins**	91 (46.2%)	7 (35.0%)	0.543
**Oral anticoagulants**	29 (14.7%)	4 (20.0%)	0.371
**Medication at discharge, *n* (%)**			
**ASA**	165 (83.8%)	13 (65.0%)	0.686
**Clopidogrel**	72 (36.5%)	7 (35.0%)	0.604
**Prasugrel**	30 (15.2%)	0 (0%)	0.289
**Ticagrelor**	56 (28.4%)	6 (30.0%)	0.474
**DAPT**	140 (71.1%)	11 (55.0%)	0.756
**ACE inhibitors**	94 (47.7%)	10 (50.0%)	0.294
**ARB**	70 (35.5%)	4 (20.0%)	0.378
**Aldosterone inhibitors**	42 (21.3%)	4 (20.0%)	0.765
**Thiazide diuretics**	41 (20.8%)	0 (0%)	0.210
**Loop diuretics**	32 (16.2%)	7 (35.0%)	**0.017**
**Calcium channel blockers**	57 (28.9%)	0 (0%)	0.134
**Beta-blockers**	140 (71.1%)	12 (60.0%)	0.797
**Statins**	177 (89.8%)	15 (75.0%)	0.738
**Oral anticoagulants**	35 (17.8%)	4 (20.0%)	0.484
**PCI vs. CABG vs. conservative treatment, *n* (%)**			
**PCI**	148 (75.1%)	19 (95.0%)	0.128
**CABG**	4 (2.0%)	0 (0.0%)	
**Conservative treatment**	45 (22.8%)	1 (5.0%)	

NYHA = New York Heart Association; CCS (I–IV) = Canadian Cardiovascular Society; Recent MI = Incidence of MI in the last 6 months. LG: Logarithm calculated for base 10.

**Table 4:** Multivariable Cox PH regression analysis with forward variable selection and all-cause mortality as the dependent variable and clinical factors (Age, troponin I, NYHA class, baseline LVEF, LG CRP, LG CK, number of vessels involved and platelet CXCL16) as covariates.

Variable	Hazard Ratio	95% CI	*p*-Value
Age	1.11	1.05–1.17	**<0.001**
LG troponin I	1.96	1.39–2.76	**<0.001**
Platelet CXCL16	2.67	1.05–6.79	**0.039**

## Data Availability

All original data except for the identity of participating subjects in the CAD cohort, and materials, will be made available upon request to madhumita.chatterjee@med.uni-tuebingen.de, dominik.rath@med.uni-tuebingen.de.

## References

[1] Chatterjee M., Geisler T. (2016). Inflammatory Contribution of Platelets Revisited: New Players in the Arena of Inflammation. Semin. Thromb. Hemost..

[2] Weber C., Badimon L., Mach F., van der Vorst E.P.C. (2017). Therapeutic strategies for atherosclerosis and atherothrombosis: Past, present and future. Thromb. Haemost..

[3] Liberale L., Ministrini S., Carbone F., Camici G.G., Montecucco F. (2021). Cytokines as therapeutic targets for cardio- and cerebrovascular diseases. Basic Res. Cardiol..

[4] Zernecke A., Weber C. (2005). Inflammatory mediators in atherosclerotic vascular disease. Basic Res. Cardiol..

[5] Lundberg G.A., Kellin A., Samnegard A., Lundman P., Tornvall P., Dimmeler S., Zeiher A.M., Hamsten A., Hansson G.K., Eriksson P. (2005). Severity of coronary artery stenosis is associated with a polymorphism in the CXCL16/SR-PSOX gene. J. Intern. Med..

[6] Sheikine Y., Sirsjo A. (2008). CXCL16/SR-PSOX—A friend or a foe in atherosclerosis?. Atherosclerosis.

[7] Minami M., Kume N., Shimaoka T., Kataoka H., Hayashida K., Yonehara S., Kita T. (2001). Expression of scavenger receptor for phosphatidylserine and oxidized lipoprotein (SR-PSOX) in human atheroma. Ann. N. Y. Acad. Sci..

[8] Borst O., Munzer P., Gatidis S., Schmidt E.M., Schonberger T., Schmid E., Towhid S.T., Stellos K., Seizer P., May A.E. (2012). The inflammatory chemokine CXC motif ligand 16 triggers platelet activation and adhesion via CXC motif receptor 6-dependent phosphatidylinositide 3-kinase/Akt signaling. Circ. Res..

[9] Seizer P., Stellos K., Selhorst G., Kramer B.F., Lang M.R., Gawaz M., May A.E. (2011). CXCL16 is a novel scavenger receptor on platelets and is associated with acute coronary syndrome. Thromb. Haemost..

[10] Shimaoka T., Kume N., Minami M., Hayashida K., Kataoka H., Kita T., Yonehara S. (2000). Molecular cloning of a novel scavenger receptor for oxidized low density lipoprotein, SR-PSOX, on macrophages. J. Biol. Chem..

[11] Shimaoka T., Nakayama T., Fukumoto N., Kume N., Takahashi S., Yamaguchi J., Minami M., Hayashida K., Kita T., Ohsumi J. (2004). Cell surface-anchored SR-PSOX/CXC chemokine ligand 16 mediates firm adhesion of CXC chemokine receptor 6-expressing cells. J. Leukoc. Biol..

[12] Yamauchi R., Tanaka M., Kume N., Minami M., Kawamoto T., Togi K., Shimaoka T., Takahashi S., Yamaguchi J., Nishina T. (2004). Upregulation of SR-PSOX/CXCL16 and recruitment of CD8+ T cells in cardiac valves during inflammatory valvular heart disease. Arterioscler. Thromb. Vasc. Biol..

[13] Abel S., Hundhausen C., Mentlein R., Schulte A., Berkhout T.A., Broadway N., Hartmann D., Sedlacek R., Dietrich S., Muetze B. (2004). The transmembrane CXC-chemokine ligand 16 is induced by IFN-gamma and TNF-alpha and shed by the activity of the disintegrin-like metalloproteinase ADAM10. J. Immunol..

[14] Hofnagel O., Luechtenborg B., Plenz G., Robenek H. (2002). Expression of the novel scavenger receptor SR-PSOX in cultured aortic smooth muscle cells and umbilical endothelial cells. Arterioscler. Thromb. Vasc. Biol..

[15] Wagsater D., Olofsson P.S., Norgren L., Stenberg B., Sirsjo A. (2004). The chemokine and scavenger receptor CXCL16/SR-PSOX is expressed in human vascular smooth muscle cells and is induced by interferon gamma. Biochem. Biophys. Res. Commun..

[16] Wuttge D.M., Zhou X., Sheikine Y., Wagsater D., Stemme V., Hedin U., Stemme S., Hansson G.K., Sirsjo A. (2004). CXCL16/SR-PSOX is an interferon-gamma-regulated chemokine and scavenger receptor expressed in atherosclerotic lesions. Arterioscler. Thromb. Vasc. Biol..

[17] Minami M., Kume N., Shimaoka T., Kataoka H., Hayashida K., Akiyama Y., Nagata I., Ando K., Nobuyoshi M., Hanyuu M. (2001). Expression of SR-PSOX, a novel cell-surface scavenger receptor for phosphatidylserine and oxidized LDL in human atherosclerotic lesions. Arterioscler. Thromb. Vasc. Biol..

[18] Sun Y., Chang Z., Zhang S. (2008). Increased serum CXCL16 level is a marker for acute coronary syndromes. Arch. Med. Res..

[19] Lehrke M., Millington S.C., Lefterova M., Cumaranatunge R.G., Szapary P., Wilensky R., Rader D.J., Lazar M.A., Reilly M.P. (2007). CXCL16 is a marker of inflammation, atherosclerosis, and acute coronary syndromes in humans. J. Am. Coll. Cardiol..

[20] Zhou F., Wang J., Wang K., Zhu X., Pang R., Li X., Zhu G., Pan X. (2016). Serum CXCL16 as a Novel Biomarker of Coronary Artery Disease in Type 2 Diabetes Mellitus: A Pilot Study. Ann. Clin. Lab. Sci..

[21] Tan K., Lu S., Chen Y., Song X., Wu X., Jin Z., Yuan F., Zhou Y., Li H., Yang T. (2011). CXC chemokine ligand 16 as a prognostic marker in patients with intermediate coronary artery lesions: A 2-year follow-up study. Tohoku J. Exp. Med..

[22] Andersen T., Ueland T., Ghukasyan Lakic T., Akerblom A., Bertilsson M., Aukrust P., Michelsen A.E., James S.K., Becker R.C., Storey R.F. (2019). C-X-C Ligand 16 Is an Independent Predictor of Cardiovascular Death and Morbidity in Acute Coronary Syndromes. Arterioscler. Thromb. Vasc. Biol..

[23] Laugsand L.E., Asvold B.O., Vatten L.J., Janszky I., Platou C., Michelsen A.E., Arain F., Damas J.K., Aukrust P., Ueland T. (2016). Soluble CXCL16 and risk of myocardial infarction: The HUNT study in Norway. Atherosclerosis.

[24] Stellos K., Sauter R., Fahrleitner M., Grimm J., Stakos D., Emschermann F., Panagiota V., Gnerlich S., Perk A., Schonberger T. (2012). Binding of oxidized low-density lipoprotein on circulating platelets is increased in patients with acute coronary syndromes and induces platelet adhesion to vascular wall in vivo--brief report. Arterioscler. Thromb. Vasc. Biol..

[25] Chatterjee M., Rath D., Schlotterbeck J., Rheinlaender J., Walker-Allgaier B., Alnaggar N., Zdanyte M., Muller I., Borst O., Geisler T. (2017). Regulation of oxidized platelet lipidome: Implications for coronary artery disease. Eur. Heart J..

[26] Meyer Dos Santos S., Blankenbach K., Scholich K., Dorr A., Monsefi N., Keese M., Linke B., Deckmyn H., Nelson K., Harder S. (2015). Platelets from flowing blood attach to the inflammatory chemokine CXCL16 expressed in the endothelium of the human vessel wall. Thromb. Haemost..

[27] Zhao L., Wu F., Jin L., Lu T., Yang L., Pan X., Shao C., Li X., Lin Z. (2014). Serum CXCL16 as a novel marker of renal injury in type 2 diabetes mellitus. PLoS ONE.

[28] Jansson A.M., Aukrust P., Ueland T., Smith C., Omland T., Hartford M., Caidahl K. (2009). Soluble CXCL16 predicts long-term mortality in acute coronary syndromes. Circulation.

[29] Ueland T., Smedbakken L.M., Hallen J., Atar D., Januzzi J.L., Halvorsen B., Jensen J.K., Aukrust P. (2012). Soluble CXCL16 and long-term outcome in acute ischemic stroke. Atherosclerosis.

[30] Lee J.S., Chang P.Y., Zhang Y., Kizer J.R., Best L.G., Howard B.V. (2017). Triglyceride and HDL-C Dyslipidemia and Risks of Coronary Heart Disease and Ischemic Stroke by Glycemic Dysregulation Status: The Strong Heart Study. Diabetes Care.

[31] Nofer J.R., Walter M., Kehrel B., Wierwille S., Tepel M., Seedorf U., Assmann G. (1998). HDL3-mediated inhibition of thrombin-induced platelet aggregation and fibrinogen binding occurs via decreased production of phosphoinositide-derived second messengers 1,2-diacylglycerol and inositol 1,4,5-tris-phosphate. Arterioscler. Thromb. Vasc. Biol..

[32] Riddell D.R., Vinogradov D.V., Stannard A.K., Chadwick N., Owen J.S. (1999). Identification and characterization of LRP8 (apoER2) in human blood platelets. J. Lipid Res..

[33] Rath D., Chatterjee M., Borst O., Muller K., Stellos K., Mack A.F., Bongartz A., Bigalke B., Langer H., Schwab M. (2014). Expression of stromal cell-derived factor-1 receptors CXCR4 and CXCR7 on circulating platelets of patients with acute coronary syndrome and association with left ventricular functional recovery. Eur. Heart. J..

[34] Cebo M., Dittrich K., Fu X., Manke M.C., Emschermann F., Rheinlaender J., von Eysmondt H., Ferreiros N., Sudmann-Innerhofer J., Witte A. (2021). Platelet ACKR3/CXCR7 Favors Anti-Platelet Lipids over an Atherothrombotic Lipidome and Regulates Thrombo-inflammation. Blood.

[35] Rath D., Rapp V., Schwartz J., Winter S., Emschermann F., Arnold D., Rheinlaender J., Buttcher M., Strebl M., Braun M.B. (2022). Homophilic Interaction Between Transmembrane-JAM-A and Soluble JAM-A Regulates Thrombo-Inflammation: Implications for Coronary Artery Disease. JACC Basic Transl. Sci..

